# Association of Matrix Gla protein gene (rs1800801, rs1800802, rs4236) polymorphism with vascular calcification and atherosclerotic disease: a meta-analysis

**DOI:** 10.1038/s41598-017-09328-5

**Published:** 2017-08-18

**Authors:** Kaixiang Sheng, Ping Zhang, Weiqiang Lin, Jun Cheng, Jiawei Li, Jianghua Chen

**Affiliations:** 1 Kidney Disease Center, The First Affiliated Hospital, College of Medicine, Zhejiang University, Hangzhou, Zhejiang China; 2 Key Laboratory Of Nephropathy, Hangzhou, Zhejiang China; 3 Kidney Disease Immunology Laboratory, the Third Grade Laboratory, State Administration of Traditional Chinese Medicine, Hangzhou, Zhejiang China

## Abstract

Association between the MGP gene rs1800801, rs1800802, rs4236 polymorphisms and vascular calcification and atherosclerotic disease was inconsistent. To clarify precise association, we performed this meta-analysis. Medline, Embase and China Knowledge Resource Integrated Database were systematically searched through December 2016. A total of 23 case-control studies, consisting of 5280 cases and 5773 controls, were included. The overall results suggested that the -7A polymorphism was associated with an increased risk for vascular calcification and atherosclerotic disease in the recessive model (OR = 1.50, 95% CI 1.01–2.24, P = 0.045). Subgroup analyses of Caucasians showed significant associations in the allelic model, recessive model, and homozygote model: allelic model (OR = 1.19, 95% CI 1.06–1.34, P = 0.004), recessive model (OR = 1.60, 95% CI 1.26–2.03, P < 0.001), homozygote model (OR = 1.83, 95% CI 1.18–2.81, P = 0.006). Subgroup analysis of the Asian population did not demonstrate any significant associations in any of the genetic models. No significant association was found in any genetic model amongst the rs1800802 and rs4236 polymorphisms. The findings of this meta-analysis indicate that the MGP gene rs1800801 polymorphism is significantly associated with vascular calcification and atherosclerotic disease, especially in the Caucasian population.

## Introduction

Atherosclerotic disease includes coronary artery diseases, cerebrovascular disease, and peripheral arterial diseases. It is still the leading cause of morbidity and mortality worldwide^1–3^. Vascular calcification occurs as a part of the atherosclerotic process and it is an active process regulated similarly to the process of bone formation^4^. According to the current theoretical knowledge, the formation of microcalcifications causes plaque instability and is correlated with cardiovascular risk^5^. Therefore, vascular calcification is supposed to be a strong predictor of cardiovascular events independent of the traditional risk factors^6, 7^.

Matrix γ-carboxyglutamic acid Gla protein (MGP), a 10-kDa vitamin K-dependent extracellular matrix protein, has been shown to be an inhibitor of vascular calcification^8, 9^. The mechanism by which MGP inhibits vascular calcification is still unknown. According to the current understanding, MGP regulates vascular calcification by binding and inactivating bone morphogenic protein 2 and preventing the deposition of calcium phosphate in the vascular matrix^10–12^.

Over the past decade, increasing evidence has indicated that several single nucleotide polymorphisms (SNPs) of the MGP gene may play a crucial role in the susceptibility of vascular calcification and atherosclerotic disease^9^. Genes rs1800801 (G7-A), rs1800802 (T138-C), rs4236 (Thr83-Ala) were most often reported. The association between MGP gene rs1800801, rs1800802, rs4236 polymorphisms and vascular calcification and atherosclerotic disease has been discussed in several studies, but the results have been controversial^13–17^. These results were inconclusive and did not reach a consensus. Therefore, we conducted this meta-analysis in order to precisely elucidate the genetic roles for the MGP gene rs1800801, rs1800802, rs4236 polymorphisms in the process of vascular calcification and atherosclerotic disease.

## Materials and Methods

### Literature search and criteria of inclusion

This meta-analysis was performed according to the Preferred Reporting Items for Systematic Reviews and Meta-Analyses (PRISMA) criteria^18^. Relevant articles were identified by a systematic search of Medline, Embase and China Knowledge Resource Integrated (CNKI) Database from their inception to December 2016. The following search terms were used: “Matrix Gla protein”, “MGP”, “NTI”, “GIG36”, “MGLAP”, “polymorphism”, “polymorphisms”, “calcification”, “atherosclerosis”, “acute coronary syndrome”, “myocardial infarction”, “stenosis”, “ischemic stroke”, and “cerebral infarction”. Two authors independently confirmed the eligibility of articles and collated the data from the qualifying articles. The reference lists of retrieved articles were also reviewed for eligible studies. There were no language restrictions.

### Inclusion criteria

Eligible articles should meet the following criteria: (1) evaluated the association of the MGP gene (rs1800801, rs1800802, rs4236) polymorphism with vascular calcification and atherosclerotic disease, (2) studied on human beings, (3) in a case-control or nested case-control study design.

### Exclusion criteria

We excluded studies according to the following criteria: (1) unrelated to the association of MGP gene polymorphism with vascular calcification and atherosclerotic disease; (2) review articles; (3) have no control group; (4) animal studies; (5) data is missing or incomplete, and the authors could not be contacted; (6) data is duplicated.

### Data extraction and quality assessment

Relevant information was carefully extracted from all eligible articles. The following data were extracted: first author, year of publication, country of origin, ethnicity, source of controls, frequency of genotypes in cases and controls, and evidence of Hardy-Weinberg equilibrium (HWE) in controls. Two authors independently extracted the data and assessed the study quality based on the Newcastle Ottawa Scale (NOS)^19^. Any study with a score greater than 7 was considered as “high quality”. Disagreements were resolved by consensus or arbitration by a third reviewer.

### Statistical analysis

All analyses were computed in Stata software version 12 (StataCorp, College Station, TX). HWE was assessed for each SNP among controls using a χ2 test^20^. Odds ratios (ORs) and 95% confidence intervals (CIs) were used to assess the strength of associations of the MGP gene (rs1800801, rs1800802, rs4236) polymorphism with vascular calcification and atherosclerotic disease. The Z test was used to assess the significance of the ORs, and a P value < 0.05 was considered statistically significant. Heterogeneity between studies was tested through chi-square and I-square (I^2^) tests. A fixed-effects model was used if the I^2^ value was less than 50% and the p-value was greater than 0.1; otherwise a random-effects model was used. Subgroup analyses were conducted based on ethnicity and source of control. Sensitivity analyses were performed to display possible variability. Begg’s and Egger’s linear regression tests were applied to assess the potential publication bias^21, 22^.

## Results

### Characteristics of studies included in this meta-analysis

**Figure 1:**
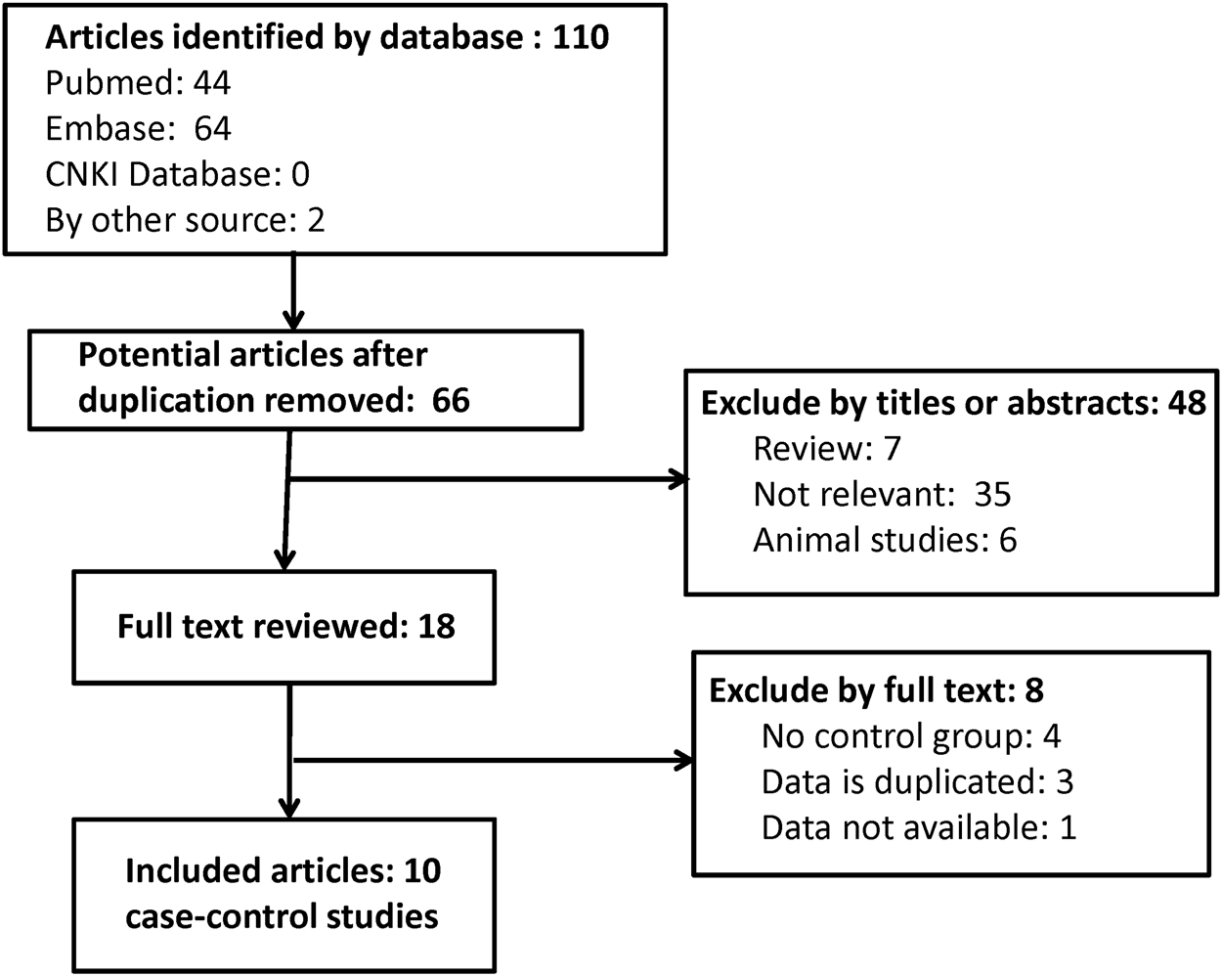
A flow diagram of selection process.

The study selection process is shown in Fig. 1. From 66 potential articles, 10 articles^13–17, 23–27^ met the inclusion criteria, including 23 studies consisting of 5280 cases and 5773 controls. Among these included studies, 18 were performed in the Caucasian population and 5 were performed in Asians. All were case-control studies and had been published between 2000 and 2016. The characteristics of eligible studies are shown in Table 1.

**Table 1:** Characteristics of studies included in this meta-analysis.

Author and year	Country	Ethnicity	Source of control	Case			Control			HWE	NOS
**rs1800802 (T-138C)**				TT	TC	CC	TT	TC	CC		
Herrmann, 2000	Belfast	Caucasian	PB	114	65	11	108	54	6	0.8137	6
Herrmann, 2000	France	Caucasian	PB	263	119	16	314	142	23	0.1872	6
Brancaccio,2005	Italy	Caucasian	HB	95	24	6	73	50	12	0.4212	8
Harbuzova,2011	Ukrainian	Caucasian	PB	68	38	9	65	40	5	0.7113	5
Harbuzova,2012	Ukrainian	Caucasian	PB	104	53	13	74	44	6	0.8684	5
Garbuzova,2012	Ukraine	Caucasian	HB	64	35	8	75	57	7	0.3567	7
Roustazadeh,2013	Iran	Asian	HB	39	33	40	34	16	20	0.0000	6
Wang,2013	China	Asian	HB	305	330	117	302	358	111	0.7681	7
Tunon-Le Poultel,2014	Spain	Caucasian	PB	52	40	12	86	77	18	0.8999	6
**rs1800801(G-7A)**				GG	GA	AA	GG	GA	AA		
Herrmann, 2000	Belfast	Caucasian	PB	85	78	29	72	82	22	0.8566	6
Herrmann, 2000	France	Caucasian	PB	143	201	69	174	247	68	0.1843	6
Herrmann, 2000	France	Caucasian	PB	10	10	2	169	179	60	0.2656	6
Brancaccio,2005	Italy	Caucasian	HB	30	63	32	47	70	18	0.3109	8
Harbuzova,2011	Ukrainian	Caucasian	PB	48	53	14	46	60	4	0.0037	5
Harbuzova,2012	Ukrainian	Caucasian	PB	61	83	26	54	62	8	0.0756	5
Garbuzova,2012	Ukraine	Caucasian	HB	48	52	14	71	63	6	0.0813	7
Wang,2013	China	Asian	HB	625	123	4	642	124	5	0.7090	7
Najafi,2014	Iran	Asian	HB	59	49	4	29	34	7	0.5146	6
**rs4236 (Thr83Ala)**				Thr/Thr	Thr/Ala	Ala/Ala	Thr/Thr	Thr/Ala	Ala/Ala		
Harbuzova,2011	Ukrainian	Caucasian	PB	49	50	16	48	51	11	0.6327	5
Harbuzova,2012	Ukrainian	Caucasian	PB	67	83	20	43	66	15	0.1753	5
Garbuzova,2012	Ukraine	Caucasian	HB	49	50	16	58	55	15	0.7232	7
Wang,2013	China	Asian	HB	584	161	7	586	173	12	0.8503	7
Ataman,2016	Ukraine	Caucasian	PB	16	19	5	13	17	10	0.3585	6

HWE, Hardy-Weinberg equilibrium; NOS, Newcastle Ottawa Scale; PB, population based; HB — hospital based.

### Meta-analysis results

Distribution and allele frequency of the three MGP gene polymorphisms in the cases and controls are shown in Table 1. The main results of this meta-analysis are presented in Table 2.

**Table 2:** Genotype distribution and allele frequency of the three MGP gene (rs1800801, rs1800802, rs4236) polymorphisms in cases and controls.

Outcome or Subgroup	Studies	Participants	Statistical Method	Effect Estimate	P value	Heterogeneity	
						I^2^	P value
**Allelic model**							
rs1800801(G-7A)	9	4438	OR(M-H, Random, 95% CI)	1.13(0.96,1.32)	0.141	50.0%	0.042
Caucasian	7	2733	OR(M-H, Fixed, 95% CI)	1.19(1.06,1.34)	0.004	33.5%	0.172
Asian	2	1705	OR(M-H, Random, 95% CI)	0.85(0.56,1.28)	0.427	60.6%	0.111
rs1800802 (T-138C)	9	4250	OR(M-H, Random, 95% CI)	0.98(0.83,1.16)	0.842	55.9%	0.02
Caucasian	7	2545	OR(M-H, Random, 95% CI)	0.93(0.75,1.14)	0.478	56.1%	0.034
Asian	2	1705	OR(M-H, Random, 95% CI)	1.17(0.78,1.77)	0.442	71.0%	0.063
HB	4	2211	OR(M-H, Random, 95% CI)	0.90(0.60,1.33)	0.590	81.7%	0.001
PB	5	2039	OR(M-H, Fixed, 95% CI)	1.03(0.89,1.20)	0.695	0.0%	0.861
rs4236 (Thr83Ala)	5	2365	OR(M-H, Fixed, 95% CI)	0.94(0.81,1.09)	0.428	0.0%	0.564
Caucasian	4	842	OR(M-H, Fixed, 95% CI)	0.98(0.80,1.20)	0.847	0.0%	0.453
HB	2	1766	OR(M-H, Fixed, 95% CI)	0.92(0.77,1.10)	0.373	4.7%	0.350
PB	3	599	OR(M-H, Fixed, 95% CI)	0.99(0.76,1.27)	0.911	0.0%	0.408
**Dominant model**							
rs1800801(G-7A)	9	4438	OR(M-H, Fixed, 95% CI)	1.06(0.92,1.22)	0.402	14.7%	0.311
Caucasian	7	2733	OR(M-H, Fixed, 95% CI)	1.12(0.95,1.33)	0.170	0.6%	0.419
Asian	2	1705	OR(M-H, Fixed, 95% CI)	0.94(0.73,1.20)	0.596	47.5%	0.168
rs1800802 (T-138C)	9	4250	OR(M-H, Random, 95% CI)	0.93(0.75,1.14)	0.457	54.7%	0.024
Caucasian	7	2545	OR(M-H, Random, 95% CI)	0.86(0.67,1.10)	0.238	54.1%	0.042
Asian	2	1705	OR(M-H, Random, 95% CI)	1.21(0.66,2.20)	0.540	72.7%	0.056
HB	4	2211	OR(M-H, Random, 95% CI)	0.83(0.50,1.38)	0.473	80.9%	0.001
PB	5	2039	OR(M-H, Fixed, 95% CI)	1.00(0.83,1.20)	0.995	0.0%	0.917
rs4236 (Thr83Ala)	5	2365	OR(M-H, Fixed, 95% CI)	0.93(0.77,1.11)	0.408	0.0%	0.865
Caucasian	4	842	OR(M-H, Fixed, 95% CI)	0.95(0.72,1.25)	0.705	0.0%	0.745
HB	2	1766	OR(M-H, Fixed, 95% CI)	0.93(0.76,1.15)	0.510	0.0%	0.665
PB	3	599	OR(M-H, Fixed, 95% CI)	0.91(0.64,1.30)	0.610	0.0%	0.501
**Recessive model**							
rs1800801(G-7A)	9	4438	OR(M-H, Random, 95% CI)	1.50(1.01,2.24)	0.045	52.6%	0.031
Caucasian	7	2733	OR(M-H, Fixed, 95% CI)	1.60(1.26,2.03)	<0.001	43.1%	0.104
Asian	2	1705	OR(M-H, Fixed, 95% CI)	0.51(0.21,1.27)	0.150	0.0%	0.335
rs1800802 (T-138C)	9	4250	OR(M-H, Fixed, 95% CI)	1.13(0.92,1.39)	0.232	0.0%	0.691
Caucasian	7	2545	OR(M-H, Fixed, 95% CI)	1.12(0.80,1.57)	0.495	0.0%	0.522
Asian	2	1705	OR(M-H, Fixed, 95% CI)	1.14(0.88,1.47)	0.325	0.0%	0.510
HB	4	2211	OR(M-H, Fixed, 95% CI)	1.10(0.86,1.40)	0.435	0.4%	0.390
PB	5	2039	OR(M-H, Fixed, 95% CI)	1.21(0.83,1.77)	0.317	0.0%	0.654
rs4236 (Thr83Ala)	5	2365	OR(M-H, Fixed, 95% CI)	0.94(0.65,1.37)	0.764	4.2%	0.383
Caucasian	4	842	OR(M-H, Fixed, 95% CI)	1.03(0.69,1.55)	0.870	0.9%	0.388
HB	2	1766	OR(M-H, Fixed, 95% CI)	0.91(0.51,1.63)	0.756	26.7%	0.243
PB	3	599	OR(M-H, Fixed, 95% CI)	0.97(0.60,1.57)	0.895	28.3%	0.248
**Homozygous model**							
rs1800801(G-7A)	9	4438	OR(M-H, Random, 95% CI)	1.50(0.95,2.38)	0.082	59.0%	0.012
Caucasian	7	2733	OR(M-H, Random, 95% CI)	1.83(1.18,2.81)	0.006	50.6%	0.059
Asian	2	1705	OR(M-H, Fixed, 95% CI)	0.48(0.19,1.20)	0.118	22.1%	0.257
rs1800802 (T-138C)	9	4250	OR(M-H, Fixed, 95% CI)	1.10(0.89,1.36)	0.395	4.5%	0.397
Caucasian	7	2545	OR(M-H, Fixed, 95% CI)	1.05(0.75,1.47)	0.781	8.7%	0.362
Asian	2	1705	OR(M-H, Fixed, 95% CI)	1.13(0.86,1.50)	0.383	41.5%	0.191
HB	4	2211	OR(M-H, Fixed, 95% CI)	1.04(0.87,1.26)	0.659	45.4%	0.139
PB	5	2039	OR(M-H, Fixed, 95% CI)	1.17(0.82,1.65)	0.391	0.0%	0.676
rs4236 (Thr83Ala)	5	2365	OR(M-H, Fixed, 95% CI)	0.91(0.61,1.35)	0.637	0.7%	0.402
Caucasian	4	842	OR(M-H, Fixed, 95% CI)	1.00(0.65,1.55)	0.985	0.0%	0.396
HB	2	1766	OR(M-H, Fixed, 95% CI)	0.91(0.50,1.65)	0.750	32.9%	0.222
PB	3	599	OR(M-H, Fixed, 95% CI)	0.91(0.54,1.53)	0.728	21.2%	0.281
**Heterozygous model**							
rs1800801(G-7A)	9	4438	OR(M-H, Fixed, 95% CI)	1.00(0.87,1.15)	0.996	0.0%	0.767
Caucasian	7	2733	OR(M-H, Fixed, 95% CI)	1.02(0.86,1.22)	0.822	0.0%	0.722
Asian	2	1705	OR(M-H, Fixed, 95% CI)	0.96(0.75,1.23)	0.755	8.7%	0.295
rs1800802 (T-138C)	9	4250	OR(M-H, Random, 95% CI)	0.88(0.72,1.08)	0.231	46.1%	0.062
Caucasian	7	2545	OR(M-H, Random, 95% CI)	0.83(0.65,1.06)	0.132	47.2%	0.078
Asian	2	1705	OR(M-H, Random, 95% CI)	1.16(0.61,2.19)	0.648	65.1%	0.090
HB	4	2211	OR(M-H, Random, 95% CI)	0.79(0.48,1.31)	0.360	76.3%	0.005
PB	5	2039	OR(M-H, Fixed, 95% CI)	0.97(0.80,1.17)	0.755	0.0%	0.902
rs4236 (Thr83Ala)	5	2365	OR(M-H, Fixed, 95% CI)	0.93(0.77,1.12)	0.465	0.0%	0.963
Caucasian	4	842	OR(M-H, Fixed, 95% CI)	0.93(0.70,1.25)	0.632	0.0%	0.896
HB	2	1766	OR(M-H, Fixed, 95% CI)	0.96(0.77,1.19)	0.694	0.0%	0.639
PB	3	599	OR(M-H, Fixed, 95% CI)	0.88(0.62,1.24)	0.459	0.0%	0.899

OR, odds ratio; CI, confidence intervals; I^2^ = I-square; PB, population based; HB — hospital based.

### A meta-analysis of the rs1800801 polymorphism with the risk of vascular calcification and atherosclerotic disease

**Figure 2:**
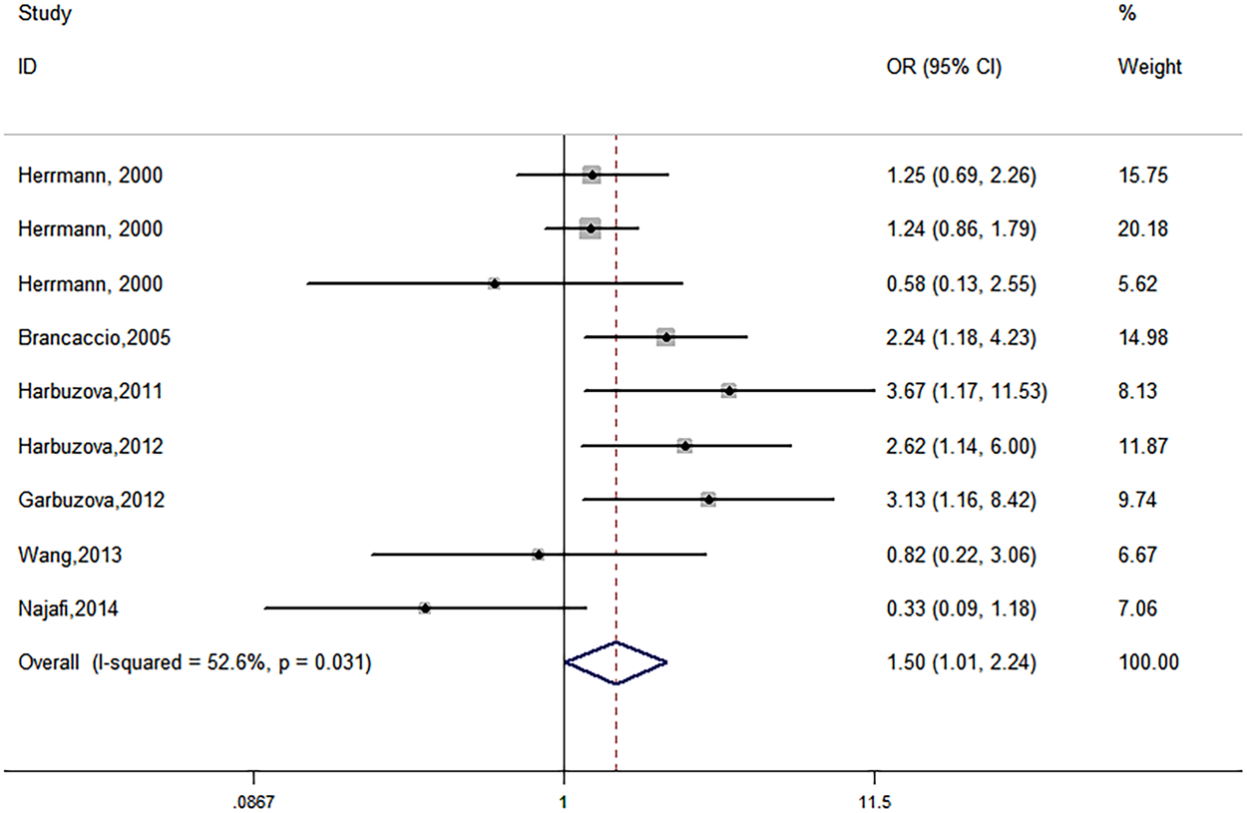
Forest plots of association of rs1800801 polymorphism with vascular calcification and atherosclerotic disease in recessive model.

Nine studies with a total of 2015 cases and 2423 controls evaluated the association of the rs1800801 polymorphism with vascular calcification and atherosclerotic disease. There was a significant association of rs1800801 gene polymorphism with vascular calcification and atherosclerotic disease in the recessive model (OR = 1.50, 95% CI 1.01–2.24, P = 0.045) (Table 2, Fig. 2). No significant association was found in the other genetic models: allelic model (OR = 1.13, 95% CI 0.96–1.32, P = 0.141), dominant model (OR = 1.06, 95% CI 0.92–1.22, P = 0.402), homozygote model (OR = 1.50, 95% CI 0.95–2.38, P = 0.082), and heterozygote model (OR = 1.00, 95% CI 0.87–1.15, P = 0.996) (Table 2).

**Figure 3:**
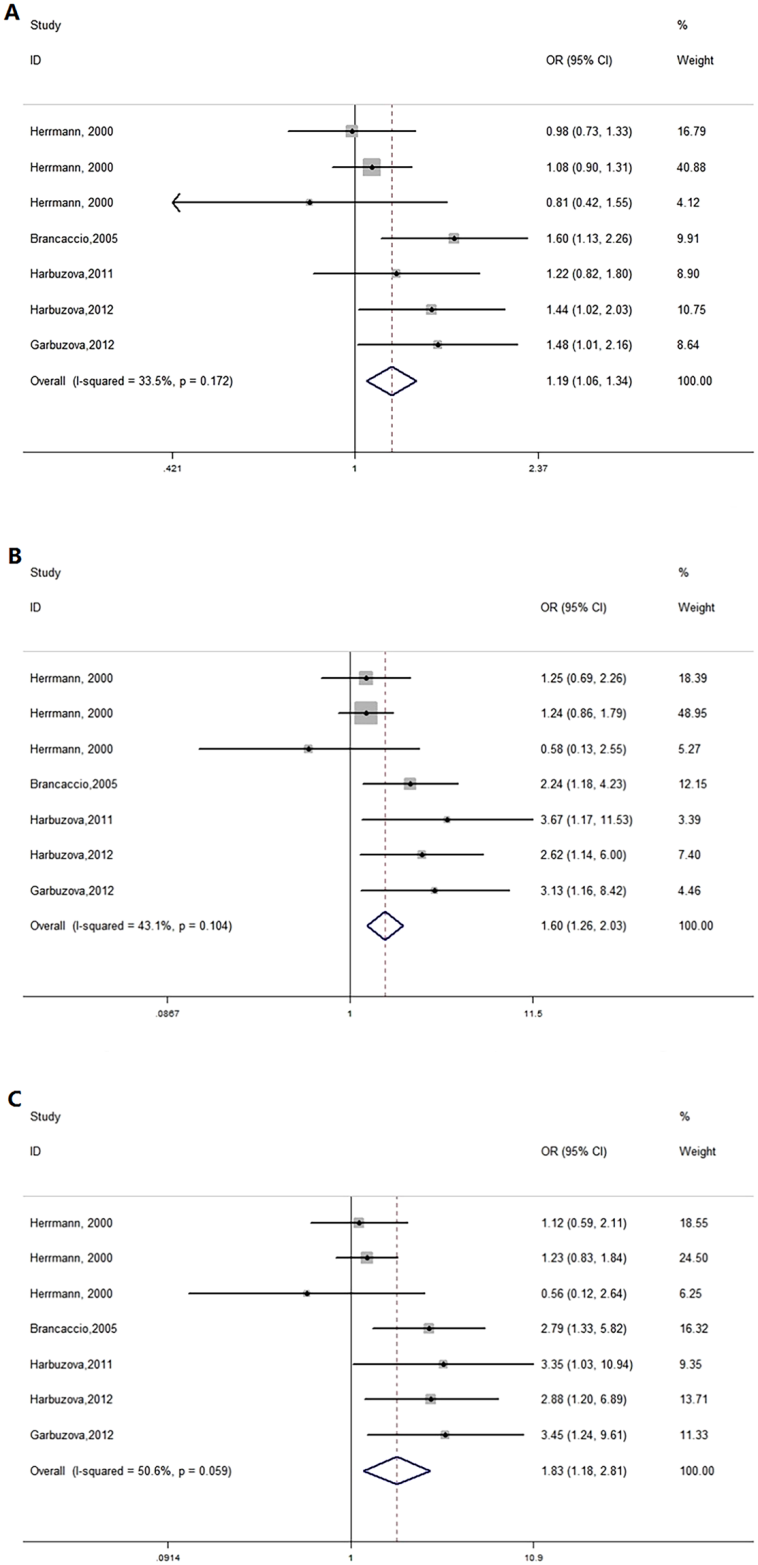
Forest plots of subgroup analyses in Caucasians, (**A**) allelic model; (**B**) recessive model; (**C**) homozygote model.

A subgroup analysis stratified by ethnicity showed a significant association amongst Caucasians in the allelic model, recessive model and homozygote model: allelic model (OR = 1.19, 95% CI 1.06–1.34, P = 0.004), dominant model (OR = 1.12, 95% CI 0.95–1.33, P = 0.170), recessive model (OR = 1.60, 95% CI 1.26–2.03, P < 0.001), homozygote model (OR = 1.83, 95% CI 1.18–2.81, P = 0.006), and heterozygote model (OR = 1.02, 95% CI 0.86–1.22, P = 0.822) (Table 2, Fig. 3). A subgroup analysis of the Asian population found no association in any genetic models: allelic model (OR = 0.85, 95% CI 0.56–1.28, P = 0.427), dominant model (OR = 0.94, 95% CI 0.73–1.20, P = 0.596), recessive model (OR = 0.51, 95% CI 0.21–1.27, P = 0.150), homozygote model (OR = 0.48, 95% CI 0.19–1.20, P = 0.118), and heterozygote model (OR = 0.96, 95% CI 0.75–1.23, P = 0.755) (Table 2).

### A meta-analysis of the association between the rs1800802 polymorphism and the risk of vascular calcification and atherosclerotic disease

Nine studies, consisting of 2073 cases and 2177 controls, evaluated the association between the rs1800802 polymorphism and vascular calcification and atherosclerotic disease. Overall, no significant association was found in any of the genetic models: allelic model (OR = 0.98, 95% CI = 0.83–1.16, P = 0.842), dominant model (OR = 0.93, 95% CI = 0.75–1.14, P = 0.457), recessive model (OR = 1.13, 95% CI = 0.92–1.39, P = 0.232), homozygote model (OR = 1.10, 95% CI = 0.89–1.36, P = 0.395), and heterozygote model (OR = 0.88, 95% CI = 0.72–1.08, P = 0.231). Similar findings were seen in the subgroup analyses in different ethnicities and source of controls (Table 2).

### A meta-analysis of rs4236 polymorphism with the risk of vascular calcification and atherosclerotic disease

Five studies, consisting of 1192 cases and 1173 controls, evaluated the association of the rs4236 polymorphism with vascular calcification and atherosclerotic disease. Overall, no significant association was found in any of the genetic models: allelic model (OR = 0.94, 95% CI = 0.81–1.09, P = 0.428), dominant model (OR = 0.93, 95% CI = 0.77–1.11, P = 0.408), recessive model (OR = 0.94, 95% CI = 0.65–1.37, P = 0.764), homozygote model (OR = 0.91, 95% CI = 0.61–1.35, P = 0.637), and heterozygote model (OR = 0.93, 95% CI = 0.77–1.12, P = 0.465). The subgroup analyses in different ethnicities and source of controls showed similar results (Table 2).

### Sensitivity analyses

**Figure 4:**
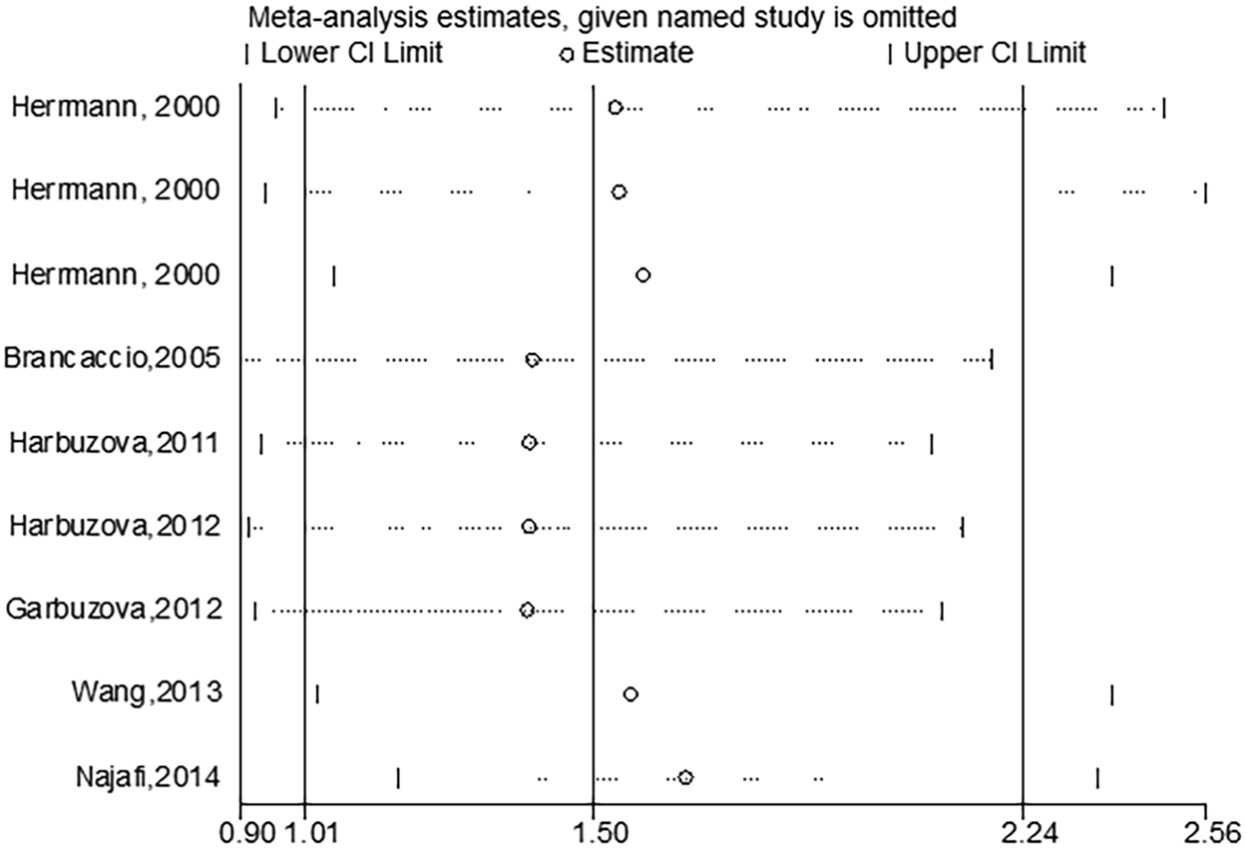
Sensitivity analysis of the rs1800801 polymorphism in recessive model.

As shown in Table 1, two studies were not consistent with the HWE in controls (P < 0.05). Hence we conducted a sensitivity analyses and observed no statistically significant changes in the pooled ORs when omitting any of the studies, which demonstrated that our results are stable and reliable (Fig. 4).

### Detection for heterogeneity

**Figure 5:**
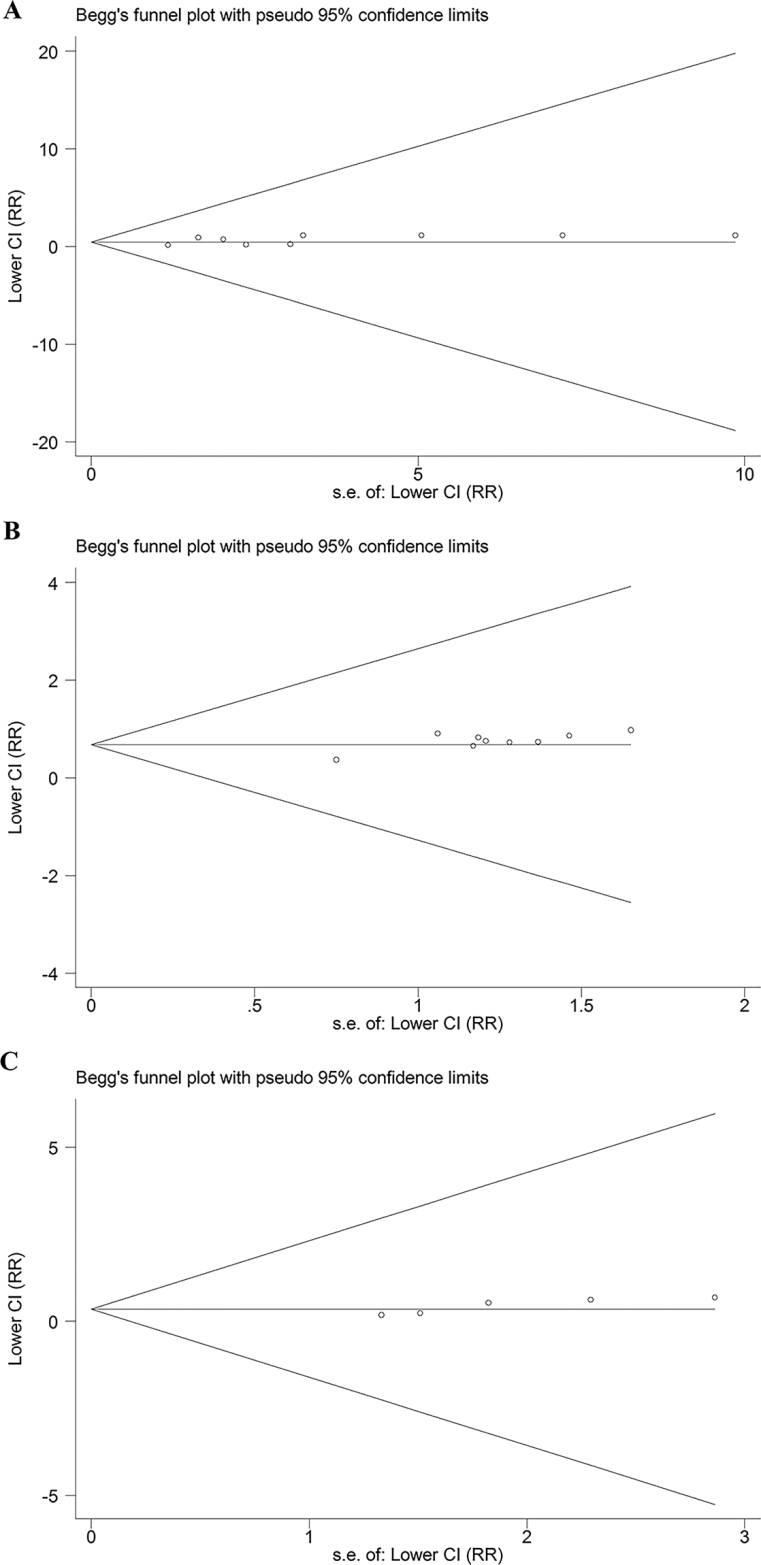
Begg’s funnel plots for assessing publication bias for (**A**) rs1800801; (**B**) rs11614913; (**C**) rs4236.

There was no significant publication bias based on the visual inspection of the funnel plots (Fig. 5). Similarly, no significant publication bias was found in the Begg’s test or Egger’s test (P > 0.05).

## Discussion

MGP is a mineral-binding extracellular matrix protein secreted by chondrocytes and vascular smooth muscle cells. It is thought to be a key regulator of vascular calcification^28^. MGP-deficient mice rapidly developed extensive vascular calcification and died due to blood vessel rupture^8^. In humans, nonsense mutations in MGP cause Keutel syndrome, a rare autosomal recessive disorder characterized by abnormal cartilage calcification^29^.

The MGP gene (NCBI-Gene ID: 4256) is located on the short arm of chromosome 12 (12p12.3). There is increasing evidence that genetic variation at the MGP locus could modulate the development of vascular calcification and atherosclerotic disease. Previous studies have shown that MGP genes rs1800801, rs1800802 and rs4236 polymorphisms have an important impact on the promoter activity^13, 26, 30^. T-138, A-7 and Ala-83 alleles of the MGP gene may contribute to the risk of vascular calcification and atherosclerotic disease, such as acute coronary syndrome and ischemic atherothrombotic stroke^13, 24, 25^. While other studies have found no significant association between these three SNPs with vascular calcification and atherosclerotic disease^14, 15, 27^. The findings have been inconsistent and inconclusive, which may be attributed to clinical heterogeneity, different ethnic populations, inadequate statistical power, and small sample sizes. Therefore, we conducted this meta-analysis and used subgroup analyses to make a more precise and convictive assessment. To our knowledge, this is the first systematic review and meta-analysis published on the association of MGP polymorphisms with vascular calcification and atherosclerotic disease.

In this meta-analysis, we investigated the association between three SNPs in the MGP gene with the risk of vascular calcification and atherosclerotic disease in 23 case-control studies (consisting of 5280 cases and 5773 controls). The overall results revealed that only the rs1800801 polymorphism was associated with the risk of vascular calcification and atherosclerotic disease. Stratification analysis by ethnicity indicated that the association was significant among Caucasians, but not among Asians in rs1800801 polymorphism. The reason why this association varies among different ethnicities is not clear, the small number of studies or the natural selection in different ethnicities may explain it. No significant association was found in the rs1800802 and rs4236 polymorphisms. Stratification analyses by ethnicity and source of control showed similar results in the rs1800802 and rs4236 polymorphisms.

The *in vitro* study revealed that the rs1800801–7A variant had an approximately 1.5-fold higher activity than -7G variant in VSMCs^30^. The -7A allele occurred more frequently in patients with vascular calcification, myocardial infarction, and ischemic atherothrombotic stroke^13, 25^. Therefore, the -7A allele of the MGP gene may confer an increased risk of vascular calcification and atherosclerotic disease, and therefore may be a novel promising target for prevention and treatment.

There are several limitations to this study. First, the association of the MGP gene polymorphism with vascular calcification and atherosclerotic disease may be influenced by gender. In some studies, the association of MGP polymorphisms with vascular calcification and atherosclerotic disease was only observed in men^13, 16, 31^. While another study found that the rs1800801 polymorphism was associated with an increased risk of ischemic atherothrombotic stroke only in women^25^. However, since detailed gender specific data could not be obtained for the studies included in this meta-analysis, we were unable to perform a sub-analysis by gender. Second, as the heterogeneity in different ethnicities influenced the results significantly, the stratification analysis of the source of control was not conducted in the SNP rs1800801. Third, only three studies were performed in Asians, therefore the findings from the Asian based studies were not convictive enough; more studies focusing on the Asian population are needed.

In conclusion, findings from this meta-analysis indicate that the MGP rs1800801 polymorphism is associated with an increased risk of vascular calcification and atherosclerotic disease. Furthermore, this association might only exist in Caucasians. The MGP gene rs1800802 and rs4236 polymorphisms are not associated with an increased risk of calcification and atherosclerotic disease. A larger number of epidemiological studies are required to confirm our findings.
